# Circulating long non-coding RNAs as diagnostic markers of lupus nephritis and 5-year follow-up disease flares

**DOI:** 10.3389/fimmu.2026.1716331

**Published:** 2026-08-04

**Authors:** Ana Flores-Chova, Olga Martinez-Arroyo, Marta Mendez-Debaets, Lesley Escriva, Ana Ortega, Maria J Forner, Raquel Cortes

**Affiliations:** 1 Cardiometabolic and Renal Risk Research Group, INCLIVA Biomedical Research Institute, Valencia, Spain; 2 CIBER of Cardiovascular diseases (CIBERCV), Madrid, Spain; 3 Internal Medicine Unit, Hospital Clinico Universitario de Valencia, Valencia, Spain; 4 Department of Medicine, University of Valencia, Valencia, Spain; 5 CIBER Diabetes and Metabolic Associated Diseases (CIBERDEM), Valencia, Spain

**Keywords:** biomarkers, diagnostic, lncRNA, lupus nephritis, prognostic, systemic lupus erythematosus

## Abstract

**Background:**

Emerging evidence underscores the pivotal role of long non-coding RNAs (lncRNAs) as epigenetic regulators in the immunopathogenesis of systemic lupus erythematosus (SLE). There is a paucity of comprehensive studies that investigate lncRNA associated with lupus nephritis (LN) and disease flares. Thus, we aimed to identify a specific circulating lncRNA panel with high diagnostic accuracy of LN and predictive power of disease flare incidence.

**Methods:**

To investigate the feasibility of early diagnosis of LN and disease flares, we examined lncRNAs present in plasma obtained from patients with SLE (*n* = 96) and healthy individuals (CNT) (*n* = 25). Non-coding RNA sequencing analysis was used to determine lncRNA in plasma, and the ability to diagnose renal damage and disease flares in a 5-year follow-up study was estimated by the area under the curve (AUC) and by using multivariate Cox’s proportional hazards regression.

**Results:**

From the ncRNA sequencing results, we identified 9,155 circulating lncRNAs and 148 differentially expressed between patients with SLE and CNT. Seven of these lncRNAs (AL139317.5, AC019080.1, LINC02185, LINC00708, AC239868.1, AC087392.4, and LATS2-AS1) presented statistically significant high expression for the discrimination of the LN (AUC ≥ 0.90), along with high specificity and positive predictive values (≥80%). A panel combining the 7-lncRNA gave an AUC that improved the read count of the single lncRNAs (1.000, *p* < 0.00001). In addition, six of these lncRNAs were associated with disease flares (AUC ≥ 0.64) and the 4-lncRNA panel was an independent predictor of disease flares (odds ratio = 8.71, *p* = 0.005 with a *c* statistic of 0.864, *p* < 0.0001), with high levels being a strong independent predictor of 60-month follow-up flares (hazard ratio 4.75, *p* = 0.005). Higher plasma 4-lncRNA panel values were also related to shorter time free of lupus flares. Finally, functional pathway analysis revealed that the lncRNA–miRNA–target gene network was linked to various inflammation pathways (MAPK, JAK-STAT, and p53 signaling pathways) in immune dysregulation and tissue damage in SLE (TGF-β signaling) and regulation of actin cytoskeleton (adherens junction and focal adhesion).

**Conclusions:**

We identified, for the first time, a specific lncRNA expression profile associated with renal damage and an increased incidence of 5-year follow-up disease flares in patients with SLE, being excellent predictive biomarkers for disease activity and organ damage.

## 1 Introduction

Systemic lupus erythematosus (SLE) is a chronic, inflammatory, and autoimmune disease characterized by the deposition of immune complexes, affecting multiple organ systems (1). Among its various manifestations, renal involvement—known as lupus nephritis (LN)—stands out as one of the most common and severe complications (2). The heterogeneous clinical presentation and unpredictable disease course of SLE pose significant challenges for early diagnosis and effective management (3).

Traditional diagnostic approaches rely on clinical criteria and serological markers, which often lack sensitivity and specificity; moreover, renal biopsy, which is an invasive method that carries potential risk and is not always feasible for continuous disease monitoring, is the gold standard for diagnosing and classifying LN (4). Therefore, there is a critical unmet need for reliable non-invasive biomarkers that not only can facilitate early and specific detection, but also support ongoing monitoring of disease activity prognostication and therapeutic strategies for SLE and LN. The lack of such tools limits the potential for truly personalized medicine in SLE care.

In recent years, accumulating evidence has highlighted the critical role of non-coding RNAs in the regulation of disease status across a wide range of pathological conditions, such as autoimmune disorders and cardiovascular and renal diseases (5–7). Among them, long non-coding RNAs (lncRNAs) are emerging as key regulators of gene expression at multiple levels, including epigenetic, transcriptional, and post-transcriptional mechanisms (8). Defined as non-protein-coding RNAs longer than 200 nucleotides, lncRNAs have gained particular attention because of their functional versatility and tissue-specific expression profiles that make them particularly suitable as disease markers. They have demonstrated significant potential as biomarkers in cardiovascular and renal pathology, including heart failure (9), hypertension (10), diabetic nephropathy (11), and acute and chronic kidney disease (12). Notably, recent studies have identified panels of circulating lncRNAs capable of distinguishing early HIV-1 infection stages (13), allograft rejection following heart transplantation (14), or lymphoblastic leukemia (15) with high sensitivity and specificity, further supporting their relevance as diagnostic biomarkers in diverse diseases.

Our group has demonstrated the alteration of circulating lncRNA levels in kidney diseases, including LN (10, 16, 17), among other authors (18, 19). These findings suggest that lncRNAs may play a crucial role in disease progression and could serve as potential non-invasive biomarkers for diagnosis and prognosis, even as therapeutic targets. Building upon this background, the present study aims to identify a specific circulating lncRNA profile in patients with SLE and to evaluate its diagnostic potential for the prediction of renal involvement and the prediction of disease flares, which remain difficult to anticipate with current clinical tools. By elucidating the role of lncRNAs in LN, we seek to contribute to the development of a set of non-invasive biomarkers that may enhance diagnosis, patient’s stratification, and more effective monitoring of disease activity in clinical practice.

## 2 Material and methods

### 2.1 Study cohort

This observational case–control study included patients with SLE and healthy volunteers from the Lupus collection provided by the INCLIVA Biobank (PT20/00029; B.000768 ISCIII), integrated in the Spanish National Biobanks Network and in the Valencian Biobanking Network. Global miRNA profiling by RNA-sequencing included 96 plasma samples from patients with SLE, 23 of whom had an LN diagnosis, and 25 healthy controls (CNT). The samples and associated clinical data were collected from follow-up visits of patients with SLE from the Internal Medicine Unit of University Clinic Hospital. Blood samples were collected for laboratory analysis, the plasma was separated by centrifugation at 2,250 × *g* for 15 min at 4 °C, aliquoted, and immediately stored at −80 °C until analysis. The patients with SLE enrolled in the present study met the American College of Rheumatology criteria for SLE. Disease activity was assessed using the Systemic Lupus Erythematosus Disease Activity Index (SLEDAI) (20). LN was determined by renal biopsy. Glomerular lesions in LN biopsies were defined according to the ISN/RPS 2003 classification, revised in 2018 (21). Healthy control subjects were age- and sex-matched to patients with SLE.

### 2.2 Follow-up

To analyze the predictive role of lncRNA for lupus flares, plasma levels were quantified at basal stage and patients were prospectively followed up during a 60-month period. Clinical and laboratory data collection and lupus flares were noted. A lupus flare was defined as worsening in SLEDAI > 3 points at the previous visit. A definition of LN flare was an increase by > 2 g/24 h proteinuria or at increment > 20% of serum creatinine of the previous state, or active urinary sediment (increase of hematuria or reappearance or increase of cellular casts) (22).

### 2.3 RNA extraction

RNA isolation and RNA sequencing were performed as described by Flores-Chova et al. (17). Total RNA was extracted from plasma samples using the miRNeasy Mini Kit (Qiagen, Hilden, Germany) from 200 μL of plasma following the protocol and instructions provided by the manufacturer. Total RNA, integrity, and quality were determined by capillary electrophoresis (Agilent 2100 Bioanalyzer, Agilent Technologies, CA, USA) with the RNA 6000 Pico chip. To normalize the protocol variability, two spike-ins (cel-miR-39 and ath-miR-159a) were added to all samples.

### 2.4 Small RNA library preparation and RNA-sequencing

Single patient libraries were prepared from 6 μL of total RNA from each sample using the Small RNA-Seq Library Prep Kit (Lexogen GmbH, Austria) following a small RNA library preparation protocol optimized to very low input samples (23). Briefly, after adapter ligation and cDNA amplification, libraries were size-selected (122- to 200-bp fragments) on a 3% agarose gel using Pippin Prep Automated DNA Size Selection platform (Sage Science, USA) and were purified, concentrated, and quantified by quantitative polymerase chain reaction (qPCR). The quality of individual libraries prior to quantification was analyzed by capillary electrophoresis in the QIAxcel Advanced System (Qiagen, Germany). Finally, single libraries were normalized to 4 nM prior to pooling and sequencing on the NextSeq 550 platform (Illumina, USA), a 150-cycle paired-end reading mode.

For small RNA sequencing (smallRNA-seq), quality control of raw data was performed using FastQC, and adapter trimming was done with TrimGalore!. The resulting trimmed reads were aligned using STAR (v.2.5.0) against the last version of the human genome (GRCh38) (24). The mature transcripts were obtained using the featureCounts function from the Rsubread package (v2.4.2, Bioconductor v3.12) (25), using lncRNA transcript annotations from Ensembl. Differential expression analysis of small RNAs was performed using the DESeq2 package (26). The raw RNA-seq dataset is available in the BioProject repository under accession number PRJNA904396.

### 2.5 Quantitative real-time PCR validation

**Figure 1:**
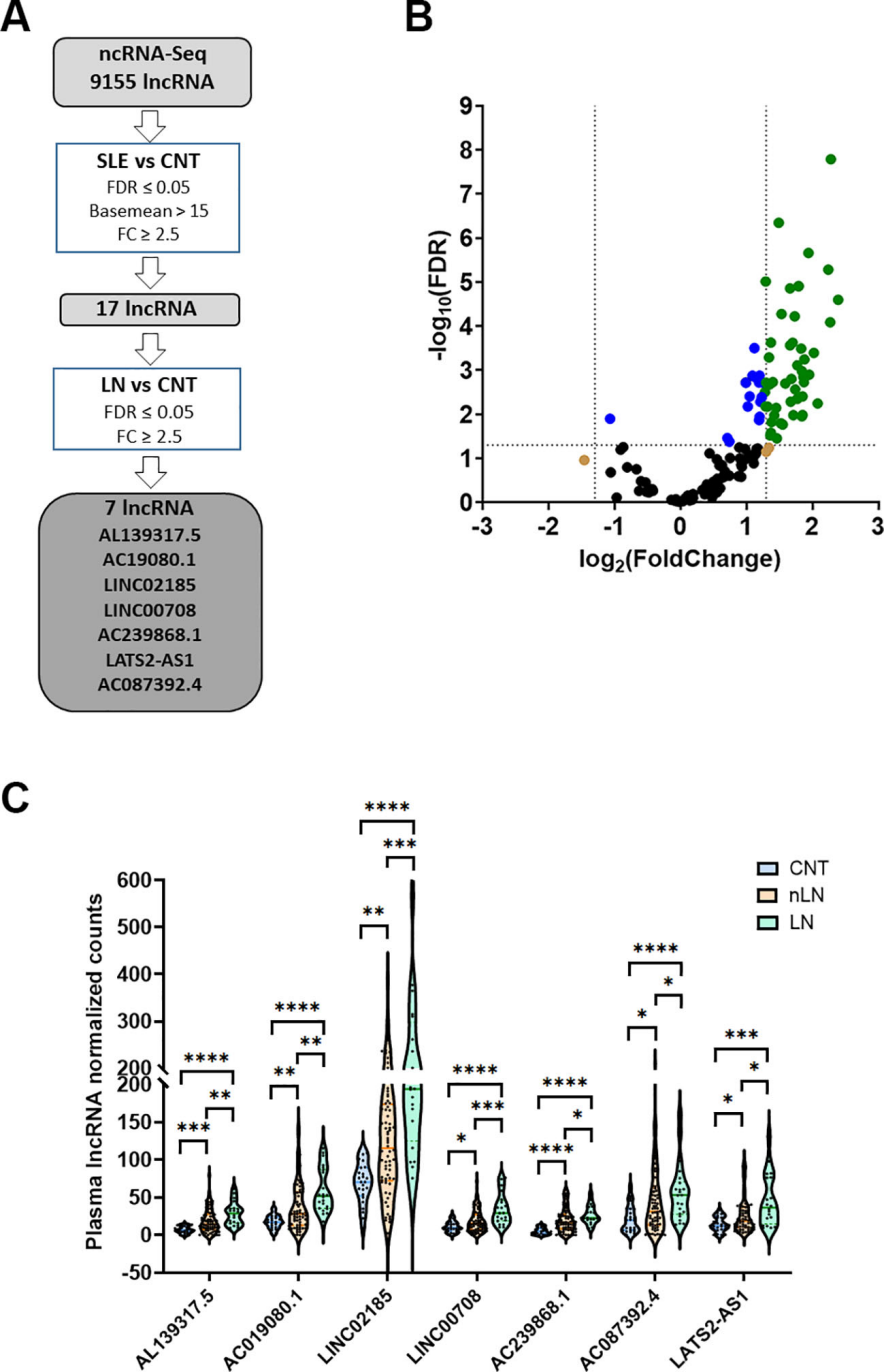
LncRNA profile in patients with SLE and LN. **(A)** Schematic overview of identification strategy of candidate long non-coding RNAs (lncRNAs) for the detection of systemic lupus erythematosus (SLE, *n* = 96) and lupus nephritis (LN, *n* = 23). FC, fold change. FDR, false discovery rate. **(B)** Volcano plot depicts the differential expressed lncRNA when comparing patients with SLE with the control group (*n* = 25). Log_2_FC, logarithm base 2 fold change. **(C)** Violin plot graph of circulating expression levels of lncRNAs selected as potential biomarkers for the detection of SLE and LN. Comparison between patients with (LN, *n* = 23) or without lupus nephritis (nLN, *n* = 73) and healthy controls (CNT, *n* = 25). Data were represented as the median (line), quartiles (dotted lines), maximum and minimum, and the probability density of the data at different values. Data were compared using Mann–Whitney *U* test. **p < 0.01, ***p < 0.001, ****p < 0.0001.

Quantitative real-time PCR (qRT-PCR) was performed to validate the expression levels of selected lncRNA identified by RNA sequencing on the LightCycler 480 II real-time PCR system (Roche, Mannheim, Germany). Candidate lncRNAs were selected based on the criteria of **Figure 1A**, a BaseMean value greater than 15 reads, a false discovery rate (FDR) ≤ 0.05, and an absolute fold change (FC) ≥ 2.5. Complementary DNA (cDNA) was synthesized from 2 μL of total RNA using the High-Capacity cDNA Reverse Transcription Kit (Applied Biosystems, USA), following the manufacturer’s instructions. A total of seven lncRNAs were included in the validation phase. Specific primers were designed by Primer 3 software (**Supplementary Table 1**). A mix of each primer set with Qiagen Multiplex PCR Master Mix and LC Green reagent (Qiagen, Hilden, Germany) was added to 2 μL of cDNA. All assays were performed in duplicate, including appropriate control and a non-template control. Additionally, melting curve analysis was conducted to assess the specificity of the generated amplicons. β-Actin (ACTB) served as a housekeeping gene for lncRNA normalization, and the relative expression of each lncRNA was calculated using the 2^−ΔΔCt^ comparative method (where Ct represents the threshold cycle). Results were expressed as FC, comparing the lncRNA levels to the average expression of the housekeeping gene.

### 2.6 Non-coding RNA target predictions and pathway enrichment analysis

Studies have shown that lncRNAs can sponge microRNAs (miRNAs), thereby influencing normal physiological processes and regulating mRNA expression. The miRNA targets for lncRNAs were predicted using DIANA-LncBase (v3) (27). In addition, to validate the predicted miRNAs, the correlation between lncRNAs and miRNAs was calculated using Spearman’s value with the normalized counts obtained with featureCounts function available in Bioconductor (v.3.12) R package Rsubread (v.2.4.2) (25) from the RNA-seq of the same SLE plasma samples. The threshold of −0.50 was used for a significant correlation between RNA interactions. Kyoto Encyclopedia of genes and genomes (KEGG) pathway analysis based on miRNA associated to lncRNA signature was performed with miRNet (28) for all the identified miRNAs together for each lncRNA. Targets were selected according to the highest FDR value. We manually screened for pathways involved in TGF-β signaling, focal adhesion, adherens junction, and regulation of the actin cytoskeleton, which are interconnected pathways that play critical roles in immune dysregulation and tissue damage in SLE. In addition, several signaling pathways were included in selected screening for its involvement in processes such as inflammation, immunity, and tissue damage (MAPK, JAK-STAT, and p53 pathways). Targets were grouped together for lncRNA, and analysis of networks was conducted using the software tool String v.12.0 (https://string-db.org/) (29). The STRING database provides a confidence score (from 0 to 1) that estimates the likelihood that an annotated interaction between a pair of proteins is biologically meaningful, specific, and reproducible. All biological interactions with a confidence score of 0.4 or higher were included.

### 2.7 Statistical analysis

Normality for each variable was evaluated using the Kolmogorov–Smirnov method. Continuous variables were expressed as median and interquartile range (non-normal distribution) or the mean ± standard deviation (normal distribution), and categorical variables were expressed as a percentage. Frequencies of qualitative variables were compared between groups using chi-square analysis. Median and mean values of quantitative variables were compared with the non-parametric Mann–Whitney *U* test and Student’s *t* test, respectively. The Bioconductor DESeq2 package (v.1.30.0) for the R software was applied for differential expression analysis (24). Contrasts between SLE groups were determined by performing a negative binomial generalized log-linear model to analyze the read counts for each gene, adjusted for age and gender. Raw *p*-values were adjusted using the Benjamini–Hochberg procedure. A log2 FC of ±1.3 was used as an appropriate threshold and an FDR cutoff value of 0.05 was considered statistically significant, along with a BaseMean > 15, to avoid the identification of false positives across the differential expression data. We performed a Spearman correlation analysis between the expression levels of each of the seven candidate lncRNAs and key disease-related parameters, including C3, C4, anti-dsDNA antibodies, and the SLEDAI and kidney-related parameters, such as UAE/urinary creatinine excretion. The power of plasma lncRNA to discriminate patients with SLE or with LN was analyzed by *c*-statistics through the receiver operating characteristic (ROC) curve. For each lncRNA, the area under the curve (AUC) and its 95% confidence interval (CI) was calculated, and *p* < 0.05 was considered statistically significant. A linear combination of 7-lncRNAs was created using logistic regression to obtain the associated probabilities for each patient. We constructed the ROC curve using these probabilities to evaluate the model diagnostic capability to LN. For discriminating disease flare presence, the AUC for each lncRNA was calculated and a panel of 4-lncRNA was created. To determine whether lncRNA panel was an independent predictor for lupus flares, binary logistic regression analyses were performed with all relevant variables included in the model (age, gender, C3, C4, log dsDNA levels, SLEDAI and treatment). LncRNA panel was introduced into the model to assess the incidence of lupus flares, as a dichotomous variable, according to the optimum cutoff point obtained from the ROC curve. To analyze time-to-event data (lupus flare), the Kaplan–Meier curve was used, representing the survival rate, and the log rank test provided a statistical comparison of two groups (> or < cutoff lncRNA panel). Finally, in the follow-up study, the estimated hazard ratio (HR) and 95% CI of lncRNA panel for flare incidence was calculated by using weighted multivariate Cox’s proportional hazards regression with a robust variance specification to obtain representative estimates. Statistical analyses were performed using SPSS software (version 20.0, IBM SPSS Inc., US) and GraphPad Prism software (GraphPad Prism 6.0, US). A *p*-value < 0.05 was considered to be statistically significant.

## 3 Results

### 3.1 Clinical characteristics of the study cohort

The study cohort included 96 plasma samples from patients with SLE, 23 of whom had an LN diagnosis, and 25 healthy controls. General patient characteristics are shown in **Table 1**. Pathological groups and controls were matched for age and sex percentage. Patients with LN had augmented levels of the double-strand DNA (dsDNA) and SLEDAI (*p* < 0.01). In addition, altered renal function in LN was observed, as indicated by low eGFR values and high proteinuria levels (*p* < 0.01). Patients with LN displayed significant differences between specific clinical characteristics compared to non-LN (nLN) groups, and they showed higher SLEDAI, worse renal function, higher incidence of immunosuppression, and higher cure rate of monoclonal antibody therapy.

**Table 1:** Patient characteristics of the systemic lupus erythematosus cohort.

Variables	Non-LN (*n* = 73)	LN (*n* = 23)	CNT (*n* = 25)
Age, years	51.9 ± 11.8	48.7 ± 9.1	40.2 ± 12.0
Female sex (%)	82.6	87.2	60.7
Serum creatinine (mg/dL)	0.7 ± 0.2	0.8 ± 0.3	0.7 ± 0.1
eGFR (CKD-EPI)	98.2 ± 14.4	84.5 ± 30.1**	105.9 ± 12.5
Log urinary albumin/urinary creatinine	0.62 (0.39–0.89)	2.12 (1.88–2.48)**	0.58 (0.44–0.72)
dsDNA (U/mL)	69.0 ± 92.6	200.7 ± 289.2**	–
ANA	8.6 ± 4.7	8.2 ± 5.3	–
C3 (mg/dL)	100.1 ± 26.3	87.6 ± 29.6	–
C4 (mg/dL)	20.8 ± 12.4	16.2 ± 10.5	–
SLEDAI	7.3 ± 5.3	14.3 ± 6.6*	–
Baseline treatment (%)			
Hydroxychloroquine	68.5	56.5	–
Azathioprine	19.2	17.4	–
Prednisone	38.4	47.8	–
Deflazacort	12.3	4.3	–
Belimumab	4.1	13.0*	–
Methotrexate	6.8	4.3	–
Mycophenolate mofetil	2.7	13.0*	–
Renal biopsy (ISN/RPS) (%)			
I	–	–	–
II	–	–	–
III	–	17.4	–
IV	–	34.8	–
V	–	17.4	–
Chronicity index	–	3.3 ± 1.4	–
Glomerular sclerosis (%)	–	56.5	–
Fibrous crescents (%)	–	12.5	–
Tubular atrophy (%)	–	56.3	–
Interstitial fibrosis (%)	–	56.3	–

SLE, systemic lupus erythematosus; LN, lupus nephropathy; CNT, control; eGFR, estimated glomerular filtration rate; dsDNA, double-stranded DNA; ANA, antinuclear antibodies; SLEDAI, Systemic Lupus Erythematosus Disease Activity Index. *p < 0.05; ** *p* < 0.01. Continuous variables are presented as mean ± standard deviation. Urinary albumin/urinary creatinine are presented as median and interquartile range and categorical variables as number of patients (*n*) and percentage (%).

Differentially expressed plasma lncRNAs from patients with systemic lupus and controls.

Specifically, 9,155 circulating lncRNAs were detected (**Figure 1A**). First, we performed a pairwise comparison adjusted by gender and age in the 121 individual samples tested, and identified 148 differentially expressed (DE) lncRNAs between patients with SLE and the control group. A volcano plot showed that all of the DE lncRNAs were upregulated in SLE group (**Figure 1B**). Then, we established a series of criteria for selecting the lncRNAs with the most potential as possible biomarkers of SLE presence (FDR < 0.05, FC ≥ 2.5, and a BaseMean > 15, indicating that these lncRNAs might be abundant and easily detected in plasma), obtaining 17 candidates. Seven of these lncRNAs (AL139317.5, AC019080.1, LINC02185, LINC00708, AC239868.1, AC087392.4, and LATS2-AS1) presented statistically significant expression (FDR < 0.05 and FC ≥ 2.5) for the discrimination of the LN presence. In addition, lncRNA expression levels were increased in patients with SLE, with higher expression observed in those with renal involvement (**Figure 1C**). Although a certain degree of overlap was present between LN and non-LN patients, seven lncRNAs, namely, AL139317.5, AC019080.1, LINC02185, LINC00708, AC239868.1, AC087392.4, and LATS2-AS1, showed statistically significant differences at the group level (FDR < 0.05 and FC ≥ 2.5), supporting their association with renal damage. In addition, lncRNA expression levels significantly increased in patients with SLE, and generally in those with renal damage (**Figure 1C**), all of which with a twofold increase, at least, compared to the control group. Finally, we performed a Spearman correlation analysis between the expression levels of each of the seven candidate lncRNAs and key disease-related parameters, including C3, C4, and anti-dsDNA antibodies, and the SLEDAI and kidney-related parameters, such as UAE/urinary creatinine excretion. This analysis showed that only selected lncRNAs were significantly associated with conventional disease activity markers. Specifically, LINC02185 showed an inverse association with both C3 levels and the SLEDAI. In addition, LINC00708 and AC239868.1 also showed significant associations with these parameters, and LATS2-AS1 showed a significant correlation with anti-dsDNA antibody levels. In addition, all lncRNA showed a significant correlation with UAE/urinary creatinine levels. The complete correlation analysis has been incorporated into the **Supplementary Material** as **Supplementary Table 2**.

### 3.2 Discriminatory power of the renal damage by the lncRNA profile

**Figure 2:**
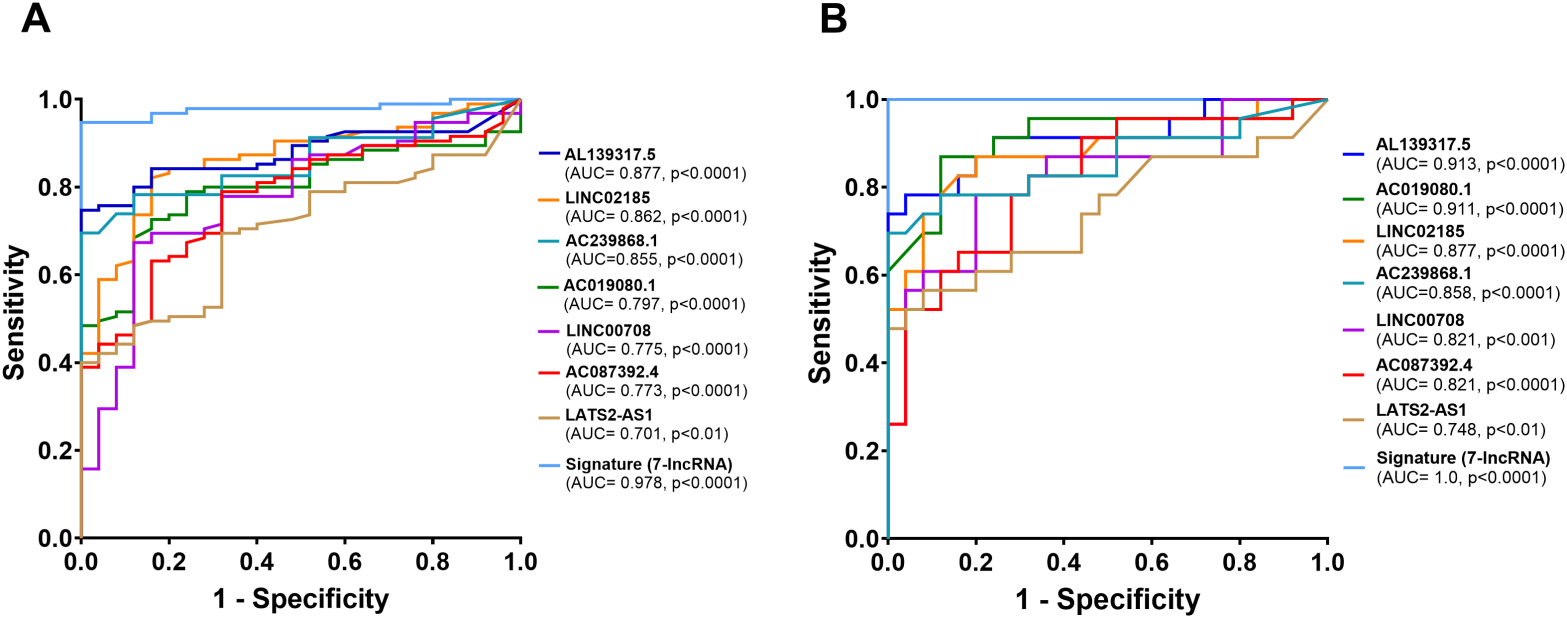
Diagnostic value of lncRNA profile in patients with SLE and LN. **(A)** Receiver operating characteristic (ROC) curves of individual plasma lncRNAs and the combined 7-lncRNA panel for the detection of SLE. AUC, area under the curve. **(B)** ROC curves of individual plasma lncRNAs and the combined 7-lncRNA panel for the detection of renal involvement (lupus nephritis, LN). AUC, area under the curve.

Since our primary objective was to identify potential biomarkers for the detection of LN, we first focused on the seven lncRNAs that were DE in patients with SLE. We subsequently evaluated their ability to discriminate patients with LN from those without renal involvement and validated the selected candidates by qRT-PCR. Importantly, the validated lncRNAs retained the statistically significant differences observed between the SLE and healthy control groups (**Supplementary Figure 1**). ROC curves were applied to analyze the capability of the altered lncRNAs to detect SLE and renal damage (**Figure 2**). All of them show relevant diagnostic capacity with at least AUC ≥ 0.77 (*p* < 0.0001) (**Figure 2A**) and AUC ≥ 0.82 (*p* < 0.0001) (**Figure 2B**) respectively, with the exception of LATS2-AS1, which shows slightly lower values, for discriminating SLE and renal damage, respectively. A panel combining the 7-lncRNA with statistically significant ROC curves in each pathological condition gave an AUC that improved the read count of the single lncRNAs (0.980 and 1.000, *p* < 0.00001, respectively). Additionally, we showed high specificity and positive predictive values for these plasma lncRNAs for SLE and LN (**Supplementary Tables 3**, **S4**, respectively). When comparing the AUC obtained from 7-lncRNA with well-established clinical markers such as UAE for LN, a significant difference was observed among the resulting models (**Supplementary Figure 2**). Finally, we performed a direct statistical comparison of the AUCs obtained for the combined signature versus each individual lncRNA. The use of the multi-lncRNA signature results in a statistically significant improvement in the discriminative performance for both SLE and LN compared with the use of individual lncRNAs. Although some single lncRNAs exhibited relatively high AUC values, the combined panel consistently achieved superior performance (**Supplementary Table 5**). In addition, when comparing the successive AUCs obtained from the different lncRNA signatures by sequentially removing the lncRNAs with the lowest AUCs, significant differences were observed among the resulting models for the SLE and LN discrimination. These findings suggest that some degree of simplification may be feasible for SLE and LN discrimination with using a 4-lncRNA panel, although a full 7-lncRNA panel provides the most robust and consistent performance, particularly for SLE diagnosis (**Supplementary Table 6**).

### 3.3 Plasma lncRNA panel association with disease flare incidence

**Figure 3:**
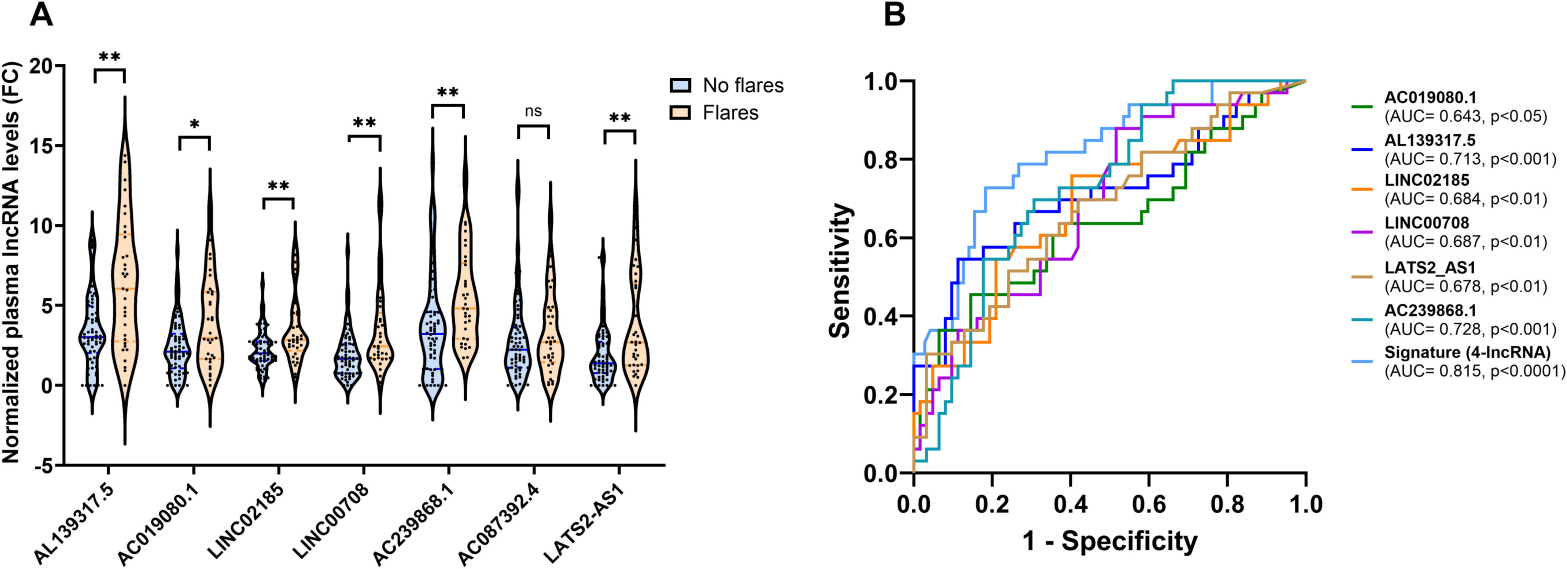
Discriminating power of plasma lncRNA for prognosis of lupus flares. **(A)** Violin plot graph of circulating expression levels of lncRNAs selected as potential biomarkers for the detection of lupus flares. Data were represented as the median (line), quartiles (dotted lines), maximum and minimum, and the probability density of the data at different values. Data were compared using Mann–Whitney *U* test. **(B)** Receiver operating characteristic curves analysis using single lncRNAs or 4-lncRNA signature to detect lupus flares in 5-year follow-up. * p < 0.05; ** p < 0.01.

**Figure 4:**
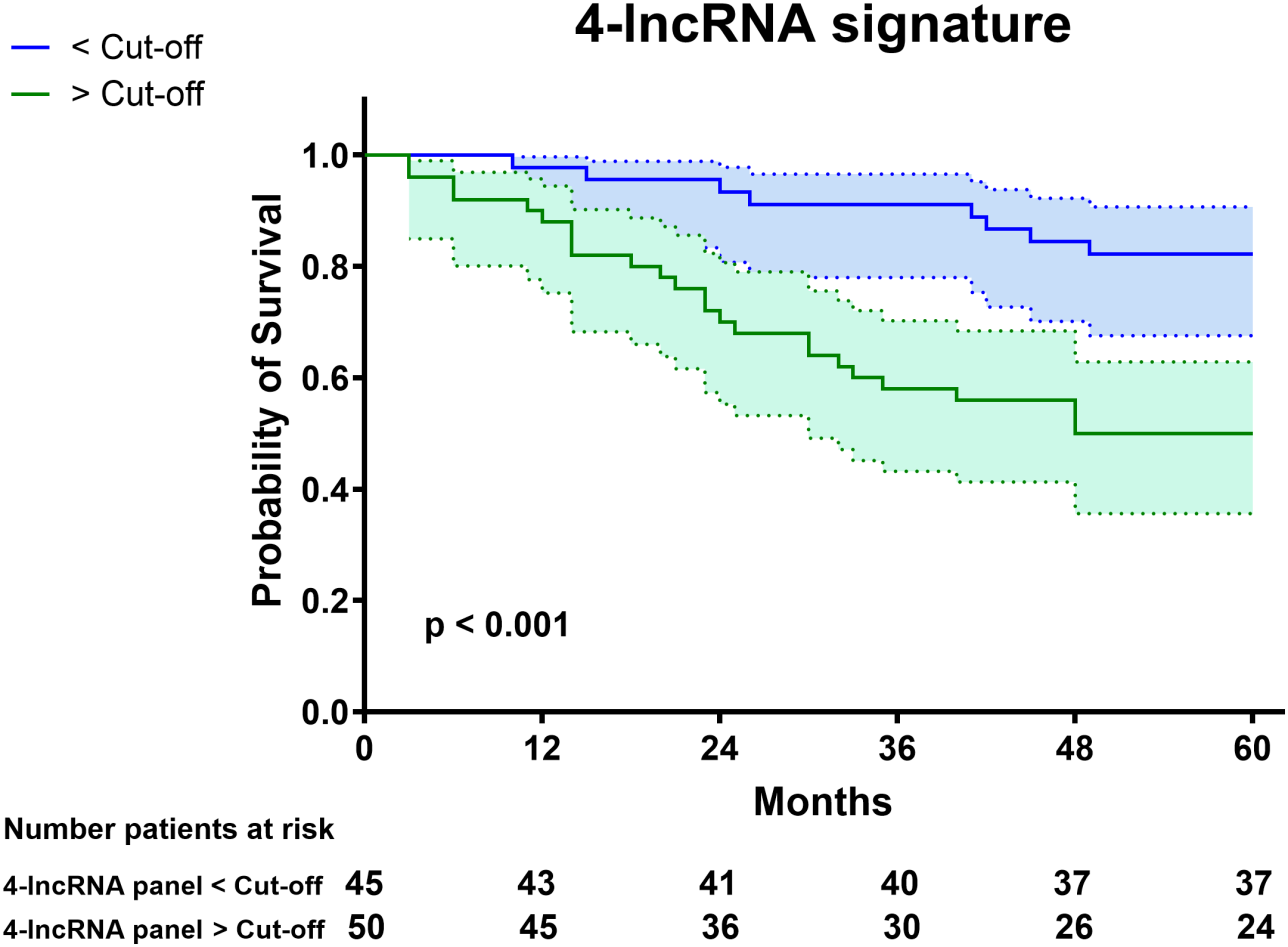
Survival in patients with SLE with specific lncRNA expression. Kaplan–Meier of lupus flare-free survival according to the 4-lncRNA signature with the cutoff obtained from receiver operating characteristic curves analysis during the follow-up period of 5-years for disease flare incidence. The samples were clustered into high expression and low expression groups of 4-lncRNA signature. Weighted long-rank test: *p*-values at the bottom survival curve is shown.

Fifty flares occurred in 34 of the 96 patients (35.4%), an average of 1.4 flares per patient, with an incidence of 0.25 flares per patient-year during the 5-year follow-up period. The median time from first studied visit to flare occurrence was 26 months. In total, 69.7% of patients had a single flare and 30.3% presented more than one. The percentage of baseline treatment for patients with or without lupus flares did not show significant differences (**Supplementary Table 7**). Thus, patients with lupus flares showed a significant ≥ 1.50-fold increase in six of the seven plasma lncRNAs (**Figure 3A**). Furthermore, lncRNAs had a significant capacity to detect flares with a minimum AUC ≥ 0.68. A panel combining the 6-lncRNA with statistically significant ROC curves gave an AUC that improved the read count of the single lncRNAs (0.853, *p* < 0.00001) (**Figure 3B**). However, when comparing the successive AUCs obtained from the different lncRNA signatures by sequentially removing the lncRNAs with the lowest AUCs, significant differences were observed among the resulting models (**Supplementary Table 8**). Based on these findings, subsequent statistical analyses for flare prediction were conducted using the 4-lncRNA signature rather than the 6-lncRNA model. Then, binary logistic regression was performed to investigate whether the plasma 4-lncRNA (AL139317.5, LINC02185, LINC00708, and AC239868.1) panel is an independent predictor of lupus flares (optimum cutoff point obtained from the ROC curve), adjusted by age, gender, SLEDAI, C4, C3, dsDNA levels, and treatment, and an odds ratio of 8.71 (95% CI 1.9–39.3, *p* = 0.005) with a *c* statistic of 0.864 (95% CI 0.79–0.94, *p* < 0.0001) was obtained. Finally, a weighted Cox proportional hazard regression model was used to estimate the HR using the cutoff value of the panel from the ROC curve, adjusted by age, gender, SLEDAI, C4, C3, dsDNA levels, treatment, disease duration, and renal organ damage. The HR for incidence of lupus flares was 4.75 (95% CI 1.60–14.09, *p* = 0.005) for the 5-year follow-up period, suggesting that subjects with SLE with a high 4-lncRNA panel have a major risk of suffering a flare within the 5 years after sampling (**Supplementary Table 9**). High plasma 4-lncRNA panel values were also individually related to shorter time free of lupus flares (**Figure 4**).

### 3.4 Differentially expressed miRNA–mRNA network from patients with lupus nephritis

**Figure 5:**
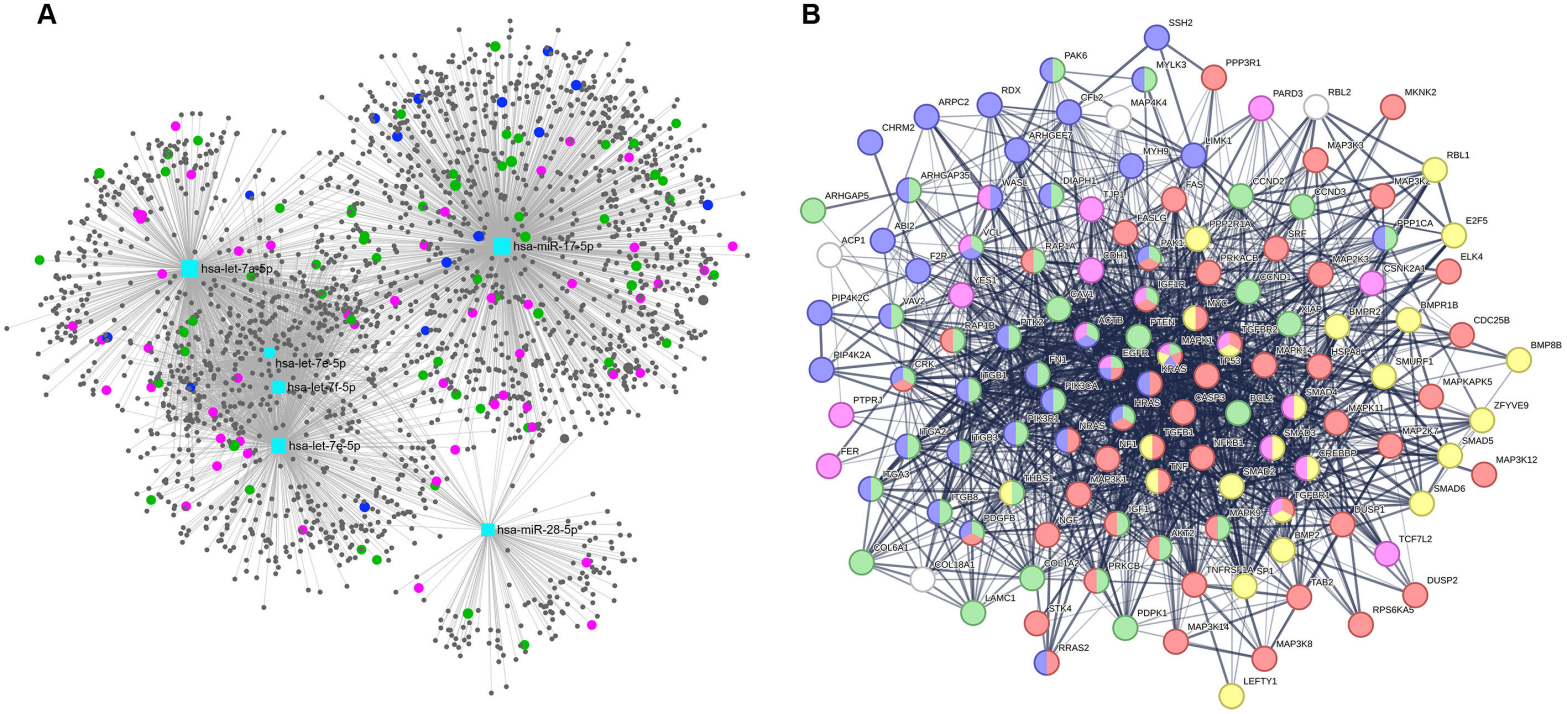
Interaction of the dysregulated miRNAs for AC239868. **(A)** Dissected miRNA–target interactions and functional associations through network‐based visual analysis (miRNet). Let-7 family, miR‐17‐5p, and miR‐28‐5p regulate 3,409 genes. Targets involved in TGF-β signaling (blue dots), MAPK signaling (purple dots), and the regulation of actin cytoskeleton (green dots) are highlighted. **(B)** Interaction of miRNA targets. STRING diagram of target protein–protein interaction of the miRNA target proteins in TGF-β signaling (yellow dots), MAPK signaling (red dots), actin cytoskeleton regulation (blue dots), focal adhesion (green dots), and adherens junction (purple dots). Lines indicate interaction evidence.

**Figure 6:**
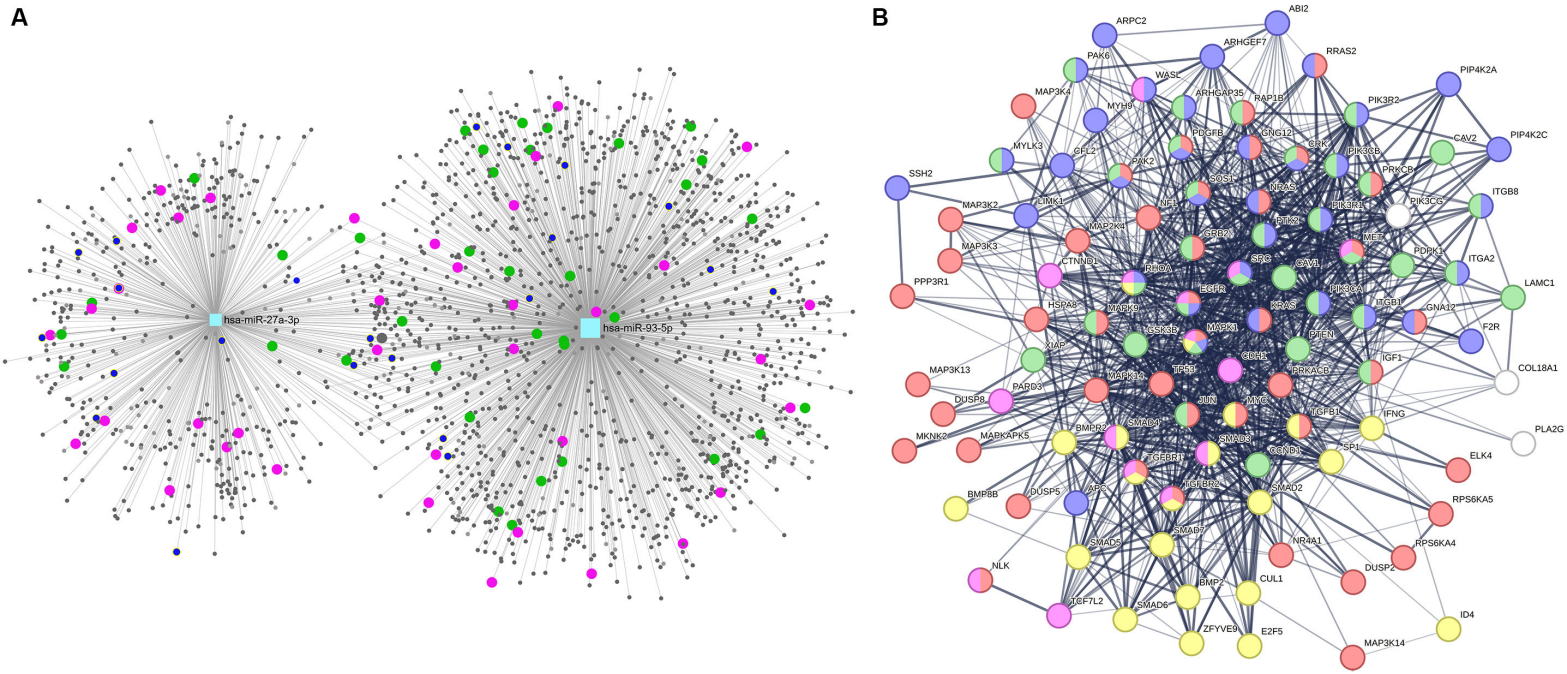
Interaction of the dysregulated miRNAs for AL019030.1. **(A)** Dissected miRNA–target interactions and functional associations through network‐based visual analysis (miRNet). MiR‐27a‐3p and miR‐93‐5p regulate 1,722 genes. Targets involved in TGF-β signaling (blue dots), MAPK signaling (purple dots), and the regulation of actin cytoskeleton (green dots) are highlighted. **(B)** Interaction of miRNA targets. STRING diagram of target protein–protein interaction of the miRNA target proteins in TGF-β signaling (yellow dots), MAPK signaling (red dots), actin cytoskeleton regulation (blue dots), focal adhesion (green dots), and adherens junction (purple dots). Lines indicate interaction evidence.

**Figure 7:**
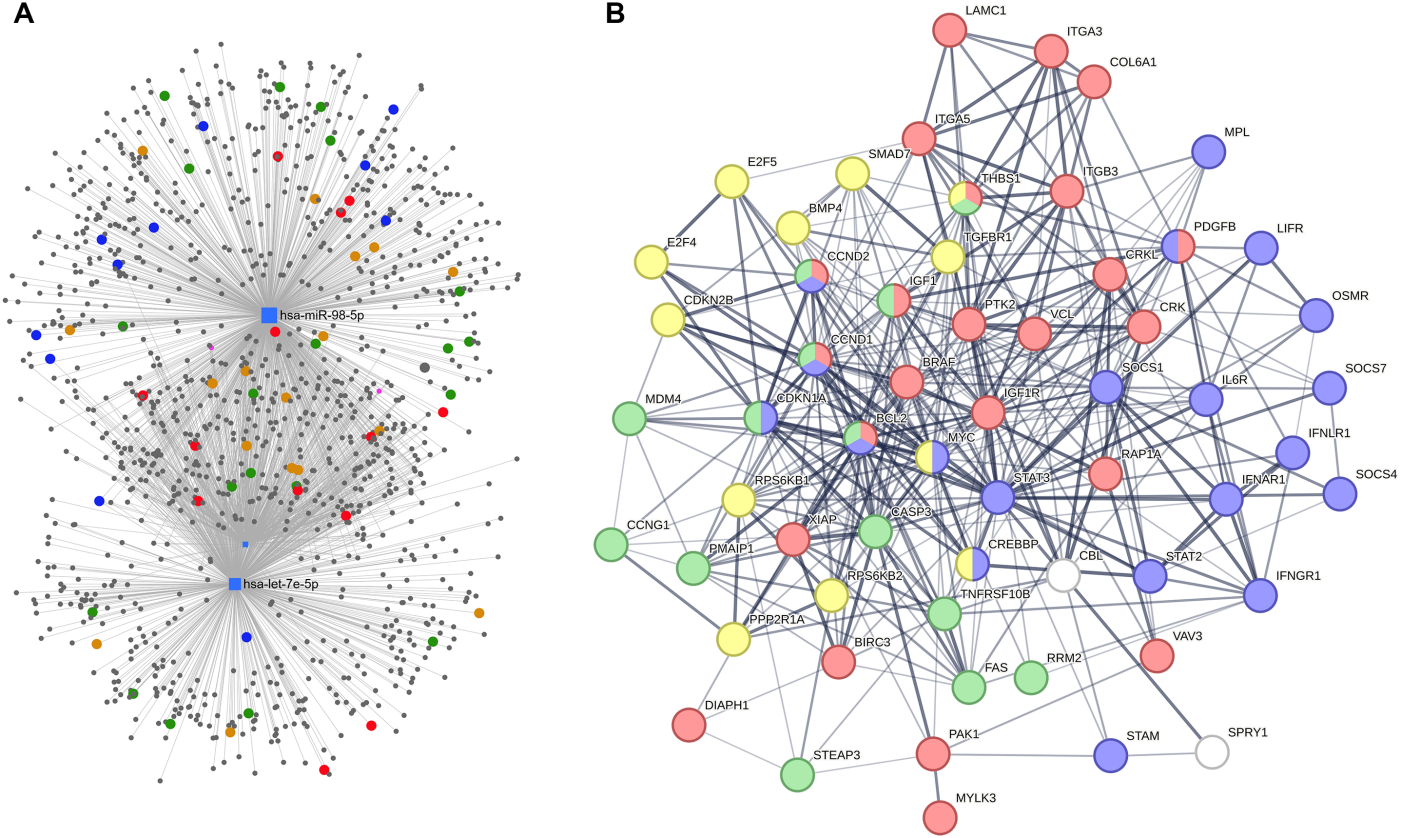
Interaction of the dysregulated miRNAs for AL139317.5. **(A)** Dissected miRNA–target interactions and functional associations through network‐based visual analysis (miRNet). miR-98-5p and let-7e-5p regulate 1,791 genes. Targets involved in TGF-β signaling (blue dots), p53 signaling (red dots), Jak-STAT signaling (orange dots), and the regulation of actin cytoskeleton (green dots) are highlighted. **(B)** Interaction of miRNA targets. STRING diagram of target protein–protein interaction of the miRNA target proteins in TGF-β signaling (yellow dots), p53 signaling (green dots), Jak-STAT signaling (blue dots), and focal adhesion (red dots). Lines indicate interaction evidence.

LncRNAs act as molecular sponges for miRNAs, preventing them from interacting with their target mRNAs, and regulating the gene expression. We examined the details of the lncRNA–miRNA interaction network in LN. The three lncRNAs identified in lncBase showed several miRNA–lncRNA interactions (**Supplementary Table 10**). In addition, we used our expression data for lncRNAs and miRNAs from RNA-seq to obtain the correlation between the miRNA identified and our three lncRNAs. Only associations with a correlation coefficient > 0.05 were included in the KEGG pathway enrichment analysis. For AL019030.1, miR-27a-3p and miR-93-5p were incorporated; for AC239868.1, miR-17-5p, miR-28-5p, and let-7 family were validated; and last, for AL139317.5, miR-98-5p, let-7e-5p, and let-7i-3p were included (**Figures 5A**, **6A**, **7A**, respectively). To identify the contribution of the miRNAs to biological processes, KEGG pathway enrichment analysis has been performed, and targets of the individual miRNAs were assessed. In total, for AC239868.1, six miRNAs (miR-17-5p, miR-28-5p, let-7a-5p, let-7e-5p, let-7f-5p, and let-7g-5p) were predicted to be involved in 60 pathways (FDR < 0.05) and targeted 3,409 genes. For AL019030.1, two miRNAs (miR-27a-3p and miR-93-5p) were predicted to be involved in 50 pathways (FDR > 0.05) and targeted 1,722 genes. Last, for AL139317.5, three miRNAs (miR-98-5p, let-7e-5p, and let-7i-3p) were predicted to be involved in 23 pathways (FDR < 0.05) and targeted 1,791 genes. Among the biological pathways regulated by the predicted miRNAs, we have highlighted those most significantly enriched and closely associated with the molecular mechanisms involved in SLE, such as TGF-β, MAPK, p53, and Jak-STAT signaling pathways and actin cytoskeleton regulation, focal adhesion, and adherens junction (**Figures 5A**, **6A**, **7A**).

Based on the KEGG analysis, we used the STRING web server to visualize the network of the protein–protein interaction of the miRNA targets for each lncRNA involved in selected pathways (**Figures 5B**, **6B**, **7B**). Network analysis showed a strong interaction of the target proteins between the pathways (PPI enrichment *p*-value < 1.0e-16). For AC239868.1 and AL019030.1, four common targets—MAPK1, EGFR, TGFBR1, and TGFBR2—are involved in at least three pathways with a high degree nodes (>35); in addition, ACTB was located in three pathways for AC239868.1 and RhoA was located in four pathways for AL019030.1; both were involved in actin cytoskeleton regulation, focal adhesion, and adherens junction, and Rho was also involved in TGF-β1 signaling. In the case of AL139317.5, four targets—THBS1, CCND1, CCND2, and BCL2—were located in three pathways; THBS1 in p53, TGF-β1 signaling, and focal adhesion, and CCND1, CCND2, and BLC2 in p53, JAK-STAT signaling, and focal adhesion.

## 4 Discussion

In recent years, increasing attention has been directed toward the study of lncRNAs due to their critical roles in the regulation of various biological processes (30). Notably, a strong association has been established between lncRNAs and several immune-mediated diseases, including SLE (31). Despite these advances, to date, no specific lncRNA signatures have been previously identified that are associated with renal involvement and the occurrence of disease flares in patients with SLE. In our study, we identified altered plasma levels of seven specific lncRNAs in patients diagnosed with SLE who also presented with renal involvement (LN). Notably, we report for the first time that elevated levels of a 4-lncRNA panel were associated with an increased incidence of disease flares during a five-year follow-up period, supporting its potential value as a prognostic biomarker signature. These findings suggest that specific lncRNA profiles may not only reflect current disease status but also predict future disease activity, offering a valuable tool for early intervention and personalized patient management.

In clinical practice, renal damage in patients with SLE is most commonly monitored using the urine albumin-to-creatinine ratio (uACR). Nevertheless, uACR lacks specificity and often fails to reflect underlying histological changes or predict long-term renal progression in LN (21, 22). The renal biopsy remains the gold standard for evaluating LN (21), providing essential diagnostic and prognostic information. However, it is an invasive procedure associated with potential complications and significant cost, which limits its use as a routine or follow-up tool. Additionally, there is a lack of specific plasma biomarkers to reliably predict SLE flares. Available markers, such as anti-dsDNA, complement C3/C4, and anti-C1q, have limited sensitivity and specificity for flare onset during routine clinical visits, leading to delayed therapeutic adjustments and often severe clinical worsening in patients (32, 33). This delay prolongs the time to remission and increases the need for intensified immunosuppression therapy and, in some cases, hospital admissions (34), thereby increasing the overall healthcare burden. In this context, the development of non-invasive, cost-effective tools, such as liquid biopsy approaches, is gaining increasing interest. The use of circulating lncRNAs as biomarkers, detectable through qPCR, presents a promising alternative (35, 36), for the dynamic monitoring of renal damage and early detection of flares in SLE, ultimately contributing to more personalized and timely disease management. Our study provides a foundational step toward addressing this clinical gap. After adjusting statistical models for these variables (C3, C4, dsDNA, and SLEDAI), the 4-lncRNA signature remained highly significant, indicating that its association with flare occurrence is not solely explained by systemic inflammation or conventional activity markers. Taken together, these findings suggest that the lncRNA signatures may provide information beyond conventional clinical and serological parameters. While we cannot completely exclude that systemic inflammation contributes to the observed lncRNA expression profile, our results support the interpretation that the signature may reflect a combination of LN-related renal damage and systemic inflammatory activity, rather than being merely a surrogate of general disease activity.

The cell type-specific expression, the stability of circulating lncRNAs in plasma, and their detectability using non-invasive sampling further support their potential for clinical application in several diseases (13–15). Prior studies have consistently linked dysregulated lncRNAs with SLE disease activity, serological markers, and LN (37–39), reinforcing our findings. However, despite being accurately detectable in the plasma of patients with SLE, the specific biological functions of these lncRNAs remain largely unknown.

Another important function of lncRNAs in the context of SLE is their ability to act as molecular sponges for miRNAs, thereby modulating post-transcriptional gene regulation. Through the competing endogenous RNA (ceRNA) mechanism, lncRNAs can bind to specific miRNAs via complementary sequences and inhibit their interaction with target mRNAs (40, 41). This regulatory mechanism can significantly influence immune-related pathways and inflammatory responses, which are critical in SLE pathogenesis. In our study, we explored this potential interaction using lncBase, a predictive tool based on experimentally validated data from miRTarBase (27), to identify miRNAs potentially targeted by the lncRNAs in our panel associated with renal damage and flare occurrence. Out of the six identified lncRNAs, only three showed potential interactions with specific miRNAs according to database predictions: AC239868 with let-7 family, miR-17-5p, and miR-28-5p; AL019080 with miR-27a-3p and miR-93-5p; and AL139317 with miR-98-5p and let-7e-5p.

These miRNAs have been implicated in the regulation of key pathogenic processes relevant to metabolic, vascular, and immune-mediated diseases. Previous studies have demonstrated that miR-28-5p is associated with metabolic syndrome and acute ischemic stroke through its modulation of HDL-C levels (42). Similarly, miR-17-5p and miR-93-5p have been linked to ischemic stroke and cerebral small vessel disease by regulating the TGF-β signaling pathway, a central mediator of vascular remodeling and fibrosis (43, 44). miR-27a-3p has been shown to regulate inflammation and immune cell infiltration by modulating macrophage polarization (45) as well as to influence apoptotic pathways (46). Notably, miR-93-5p has also been implicated in osteoarthritis and rheumatoid arthritis via targeting MMP3, in coronary atherosclerosis through modulation of cholesterol levels via ABCA1, and in chronic kidney disease by promoting renal interstitial fibrosis (47). In the context of SLE, miR-98-5p has been identified as a key regulator of dendritic cell differentiation and interferon pathway activation, promoting epigenetic changes such as methylation of the IFI44L promoter (48). Lastly, members of the let-7 family have been implicated in the pathogenesis of fibrosis across multiple organs—including the skin, lungs, and kidneys—by counteracting the pro-fibrotic actions of TGF-β (49).

Lastly, our KEGG pathway analysis revealed that the most relevant pathways associated with renal damage and flare activity linked to our lncRNA panel included the TGF-β signaling pathway, known to drive fibrosis and inflammation (50); the MAPK signaling pathway, which plays a key role in immune activation and cellular stress responses (51); the JAK-STAT pathway, essential for cell survival signaling that regulates gene expressions related to inflammation and immunity (52); p53 signaling, which not only contributes to inflammation and the survival of autoreactive immune cells, but also has the potential to suppress autoimmunity (53); and pathways involved in actin cytoskeleton regulation, such as focal adhesions and adherens junctions (54). These cytoskeletal-related pathways are essential for maintaining cell–cell adhesion and anchoring cells to the extracellular matrix, thereby contributing to tissue architecture, intercellular signaling, and cell migration. In the context of SLE, dysregulation of these pathways may underlie key pathological processes such as immune cell infiltration, tissue remodeling, and progression of renal injury (55–58).

These findings suggest that the lncRNAs identified in our study may not only serve as biomarkers but also participate actively in the regulation of key molecular processes underlying LN and disease exacerbation.

Despite the promising results, several limitations must be considered before clinical translation. While our study broadens the panel of candidate lncRNAs, further research is needed, through a larger, independent (including other chronic kidney diseases and autoimmune disorders), and multi-center cohort to enhance the robustness and generalizability of our findings. In addition, while bioinformatic approaches provide valuable insights, the involvement of pathways such as MAPK, JAK-STAT, p53, and TGF-β is based on predictive models and requires confirmation through future functional or expression studies. Nevertheless, our results presented demonstrate sufficient scientific rigor to be considered relevant and meaningful within the current understanding of SLE pathogenesis and biomarker discovery. They provide a solid foundation for future studies aiming to validate and expand the clinical utility of lncRNA-based tools in the management of LN and incidence of disease flares.

## 5 Conclusions

In conclusion, our study identifies a plasma lncRNA signature associated with renal involvement and flare occurrence in SLE, supporting their utility as non-invasive biomarkers. These lncRNAs show potential regulatory roles through predicted interactions with SLE-related miRNAs and enrichment in key pathways such as TGF-β and MAPK signaling, and actin cytoskeleton regulation. Their involvement in immune modulation, fibrosis, and cytoskeletal regulation suggests that they are not only markers of disease activity but also functional mediators in lupus pathogenesis.

## Data Availability

The datasets presented in this study can be found in online repositories. The names of the repository/repositories and accession number(s) can be found below: https://www.ncbi.nlm.nih.gov/bioproject/?term=PRJNA904396 Accession number PRJNA904396.

## References

[1] Pan L Lu MP Wang JH Xu M Yang SR . Immunological pathogenesis and treatment of systemic lupus erythematosus. World J Pediatr. (2020) 16:19–30. doi: 10.1007/s12519-019-00229-3 30796732 PMC7040062

[2] Morales E Galindo M Trujillo H Praga M . Update on lupus nephritis: Looking for a new vision. Nephron. (2021) 145:1–13. doi: 10.1159/000511268 33147587

[3] Tsokos GC . Systemic lupus erythematosus. N Engl J Med. (2011) 365:2110–21. doi: 10.1056/NEJMra1100359 22129255

[4] Rodriguez-Ramirez S Wiegley N Mejia-Vilet JM . Kidney biopsy in management of lupus nephritis: A case-based narrative review. Kidney Med. (2023) 6:100772. doi: 10.1016/j.xkme.2023.100772 38317756 PMC10840121

[5] Esteller M . Non-coding RNAs in human disease. Nat Rev Genet. (2011) 12:861–74. doi: 10.1038/nrg3074 22094949

[6] Grau-Perez M Martinez-Arroyo O Rubia-Martinez M Flores-Chova A Rodriguez-Hernandez Z Fernández-Navarro P . Association of miR-126-3p, miR-1260b and miR-374a-5p with the incidence of heart failure in a population-based cohort: The Hortega follow-up study. Eur J Intern Med. (2025) 135:118–25. doi: 10.1016/j.ejim.2025.03.022 40155222

[7] Flores-Chova A Martinez-Arroyo O Puertes A Vela-Bernal S Gorriz JL Solis-Salguero MA . Exosomal specificity of miR-15a-5p as marker of activity, renal damage and disease flares in systemic lupus erythematosus. Am J Nephrol. (2025) 13:1–19. doi: 10.1159/000544854 40081350

[8] Quinn JJ Chang HY . Unique features of long non-coding RNA biogenesis and function. Nat Rev Genet. (2016) 17:47–62. doi: 10.1038/nrg.2015.10 26666209

[9] Caporali A Anwar M Devaux Y Katare R Martelli F Srivastava PK . Non-coding RNAs as therapeutic targets and biomarkers in ischaemic heart disease. Nat Rev Cardiol. (2024) 21:556–73. doi: 10.1038/s41569-024-01001-5 38499868

[10] Riffo-Campos AL Perez-Hernandez J Martinez-Arroyo O Ortega A Flores-Chova A Redon J . Biofluid specificity of long non-coding RNA profile in hypertension: Relevance of exosomal fraction. Int J Mol Sci. (2022) 23:5199. doi: 10.3390/ijms23095199 35563588 PMC9101961

[11] Sun SF Tang PMK Feng M Xiao J Huang XR Li P . Novel lncRNA Erbb4-IR promotes diabetic kidney injury in db/db mice by targeting miR-29b. Diabetes. (2018) 67:731–44. doi: 10.2337/db17-0816 29222368

[12] Lorenzen JM Thum T . Long noncoding RNAs in kidney and cardiovascular diseases. Nat Rev Nephrol. (2016) 12:360–73. doi: 10.1038/nrneph.2016.51 27140855

[13] Biswas S Nagarajan N Hewlett I Devadas K . Identification of a circulating long non-coding RNA signature panel in plasma as a novel biomarker for the detection of acute/early-stage HIV-1 infection. biomark Res. (2024) 12:61. doi: 10.1186/s40364-024-00597-7 38867244 PMC11167902

[14] Pérez-Carrillo L Giménez-Escamilla I González-Torrent I Delgado-Arija M Sánchez-Lázaro I García-Manzanares M . Circulating long non-coding RNAs detection after heart transplantation and its accuracy in the diagnosis of acute cardiac rejection. biomark Res. (2024) 12:49. doi: 10.1186/s40364-024-00590-0 38735964 PMC11089702

[15] Huang X Huang L Xie Q Zhang L Huang S Hong M . LncRNAs serve as novel biomarkers for diagnosis and prognosis of childhood ALL. biomark Res. (2021) 9:45. doi: 10.1186/s40364-021-00303-x 34112247 PMC8193891

[16] Riffo-Campos AL Perez-Hernandez J Ortega A Martinez-Arroyo O Flores-Chova A Redon J . Exosomal and plasma non-coding RNA signature associated with urinary albumin excretion in hypertension. Int J Mol Sci. (2022) 23:823. doi: 10.3390/ijms23020823 35055008 PMC8775608

[17] Flores-Chova A Martinez-Arroyo O Riffo-Campos AL Ortega A Forner MJ Cortes R . Plasma exosomal non-coding RNA profile associated with renal damage reveals potential therapeutic targets in lupus nephritis. Int J Mol Sci. (2023) 24:7088. doi: 10.3390/ijms24087088 37108249 PMC10139178

[18] Shen M Duan C Xie C Wang H Li Z Li B . Identification of key interferon-stimulated genes for indicating the condition of patients with systemic lupus erythematosus. Front Immunol. (2022) 13:962393. doi: 10.3389/fimmu.2022.962393 35967341 PMC9365928

[19] Szeto CC So H Poon PYK Luk CCW Ng JKC Fung WWS . Urinary long non-coding RNA levels as biomarkers of lupus nephritis. Int J Mol Sci. (2023) 24:11813. doi: 10.3390/ijms241411813 37511572 PMC10380660

[20] Floege J Barbour SJ Cattran DC Hogan JJ Nachman PH Tang SCW . Management and treatment of glomerular diseases (part 1): Conclusions from a Kidney Disease: Improving Global Outcomes (KDIGO) Controversies Conference. Kidney Int. (2019) 95:268–80. doi: 10.1016/j.kint.2018.10.018 30665568

[21] Bajema IM Wilhelmus S Alpers CE Bruijn JA Colvin RB Cook HT . Revision of the International Society of Nephrology/Renal Pathology Society classification for lupus nephritis: Clarification of definitions, and modified National Institutes of Health activity and chronicity indices. Kidney Int. (2018) 93:789–96. doi: 10.1016/j.kint.2017.11.023 29459092

[22] Petri M Genovese M Engle E Hochberg M . Definition, incidence, and clinical description of flare in systemic lupus erythematosus. A prospective cohort study. Arthritis Rheum. (1991) 34:937–44. doi: 10.1002/art.1780340802 1859487

[23] Olivares D Perez-Hernandez J Perez-Gil D Chaves FJ Redon J Cortes R . Optimization of small RNA library preparation protocol from human urinary exosomes. J Transl Med. (2020) 18:132. doi: 10.1186/s12967-020-02298-9 32188466 PMC7081560

[24] Liao Y Smyth GK Shi W . featureCounts: An efficient general purpose program for assigning sequence reads to genomic features. Bioinformatics. (2014) 30:923–30. doi: 10.1093/bioinformatics/btt656 24227677

[25] Dobin A Davis CA Schlesinger F Drenkow J Zaleski C Jha S . STAR: Ultrafast universal RNA-seq aligner. Bioinformatics. (2013) 29:15–21. doi: 10.1093/bioinformatics/bts635 23104886 PMC3530905

[26] Love MI Huber W Anders S . Moderated estimation of fold change and dispersion for RNA-seq data with DESeq2. Genome Biol. (2014) 15:550. doi: 10.1186/s13059-014-0550-8 25516281 PMC4302049

[27] Karagkouni D Paraskevopoulou MD Tastsoglou S Skoufos G Karavangeli A Pierros V . DIANA-LncBase v3: Indexing experimentally supported miRNA targets on non-coding transcripts. Nucleic Acids Res. (2020) 48:D101–10. doi: 10.1093/nar/gkz1036 31732741 PMC7145509

[28] Chang L Xia J . MicroRNA regulatory network analysis using miRNet 2.0. Methods Mol Biol. (2023) 2594:185–204. doi: 10.1007/978-1-0716-2815-7_14 36264497

[29] Szklarczyk D Kirsch R Koutrouli M Nastou K Mehryary F Hachilif R . The STRING database in 2023: Protein-protein association networks and functional enrichment analyses for any sequenced genome of interest. Nucleic Acids Res. (2023) 51:D638–46. doi: 10.1093/nar/gkac1000 36370105 PMC9825434

[30] Ransohoff JD Wei Y Khavari PA . The functions and unique features of long intergenic non-coding RNA. Nat Rev Mol Cell Biol. (2018) 19:143–57. doi: 10.1038/nrm.2017.104 29138516 PMC5889127

[31] Wu GC Pan HF Leng RX Wang DG Li XP Li XM . Emerging role of long noncoding RNAs in autoimmune diseases. Autoimmun Rev. (2015) 14:798–805. doi: 10.1016/j.autrev.2015.05.004 25989481

[32] Gensous N Marti A Barnetche T Blanco P Lazaro E Seneschal J . Predictive biological markers of systemic lupus erythematosus flares: A systematic literature review. Arthritis Res Ther. (2017) 19:238. doi: 10.1186/s13075-017-1442-6 29065901 PMC5655881

[33] Calatroni M Conte E Stella M De Liso F Reggiani F Moroni G . Clinical and immunological biomarkers can identify proliferative changes and predict renal flares in lupus nephritis. Arthritis Res Ther. (2025) 27:72. doi: 10.1186/s13075-025-03536-5 40165257 PMC11956191

[34] Palazzo L Lindblom J Mohan C Parodis I . Current insights on biomarkers in lupus nephritis: A systematic review of the literature. J Clin Med. (2022) 11:5759. doi: 10.3390/jcm11195759 36233628 PMC9570701

[35] Mihaylova G Vasilev V Kosturkova MB Stoyanov GS Radanova M . Long non-coding RNAs as new biomarkers in lupus nephritis: A connection between present and future. Cureus. (2020) 12:e9003. doi: 10.7759/cureus.9003 32775083 PMC7402529

[36] Tan G Baby B Zhou Y Wu T . Emerging molecular markers towards potential diagnostic panels for lupus. Front Immunol. (2022) 12:808839. doi: 10.3389/fimmu.2021.808839 35095896 PMC8792845

[37] Sentis G Loukogiannaki C Malissovas N Nikolopoulos D Manolakou T Flouda S . A network-based approach reveals long non-coding RNAs associated with disease activity in lupus nephritis: Key pathways for flare and potential biomarkers to be used as liquid biopsies. Front Immunol. (2023) 14:1203848. doi: 10.3389/fimmu.2023.1203848 37475860 PMC10355154

[38] Cai B Cai J Yin Z Jiang X Yao C Ma J . Long non-coding RNA expression profiles in neutrophils revealed potential biomarker for prediction of renal involvement in SLE patients. Rheumatol (Oxford). (2021) 60:1734–46. doi: 10.1093/rheumatology/keaa575 33068407

[39] Zhang LH Xiao B Zhong M Li Q Chen JY Huang JR . LncRNA NEAT1 accelerates renal mesangial cell injury via modulating the miR-146b/TRAF6/NF-κB axis in lupus nephritis. Cell Tissue Res. (2020) 382:627–38. doi: 10.1007/s00441-020-03248-z 32710276

[40] Pooresmaeil F Azadi S Hasannejad-Asl B Takamoli S Bolhassani A . Pivotal role of miRNA-lncRNA interactions in human diseases. Mol Biotechnol. (2024) 67(12):4363–85. doi: 10.1007/s12033-024-01343-y 39673006

[41] Karagkouni D Karavangeli A Paraskevopoulou MD Hatzigeorgiou AG . Characterizing miRNA-lncRNA interplay. Methods Mol Biol. (2021) 2372:243–62. doi: 10.1007/978-1-0716-1697-0_21 34417757

[42] Zhang X . Correlation analysis between high-density lipoprotein cholesterol-carried microRNA-28-5p and acute ischemic stroke. J Neurochem. (2025) 169:e70119. doi: 10.1111/jnc.70119 40536249

[43] van Kralingen JC McFall A Ord ENJ Coyle TF Bissett M McClure JD . Altered extracellular vesicle microRNA expression in ischemic stroke and small vessel disease. Transl Stroke Res. (2019) 10:495–508. doi: 10.1007/s12975-018-0682-3 30617992 PMC6733813

[44] Ramzan F D'Souza RF Durainayagam BR Milan AM Markworth JF Miranda-Soberanis V . Circulatory miRNA biomarkers of metabolic syndrome. Acta Diabetol. (2020) 57:203–14. doi: 10.1007/s00592-019-01406-6 31435783

[45] Zhao X Sun Z Xu B Duan W Chang L Lai K . Degenerated nucleus pulposus cells derived exosome carrying miR-27a-3p aggravates intervertebral disc degeneration by inducing M1 polarization of macrophages. J Nanobiotechnology. (2023) 21:317. doi: 10.1186/s12951-023-02075-y 37667246 PMC10478255

[46] Moradi Vastegani Z Ghaedi-Heidari R Oroujalian A Peymani M Ghaedi K . Manuscript title: Unravelling the neuroprotective role of miR-27a-3p in the MAPK pathway in Parkinson's disease. Metab Brain Dis. (2025) 40:141. doi: 10.1007/s11011-025-01568-z 40067516

[47] Hussen BM Abdullah SR Rasul MF Jawhar ZH Faraj GSH Kiani A . MiRNA-93: A novel signature in human disorders and drug resistance. Cell Commun Signal. (2023) 21:79. doi: 10.1186/s12964-023-01106-3 37076893 PMC10114484

[48] Huang X Tan Y Wu R Li Q Luo S . MicroRNA-98-5p inhibits IFI44L-mediated differentiation of dendritic cells and activation of interferon pathway in systemic lupus erythematosus. Immunol Invest. (2024) 53:475–89. doi: 10.1080/08820139.2023.2300346 38198612

[49] Fierro-Fernández M Miguel V Lamas S . Role of redoximiRs in fibrogenesis. Redox Biol. (2016) 7:58–66. doi: 10.1016/j.redox.2015.11.006 26654978 PMC4683389

[50] Massagué J Sheppard D . TGF-β signaling in health and disease. Cell. (2023) 186:4007–37. doi: 10.1016/j.cell.2023.07.036 37714133 PMC10772989

[51] Cargnello M Roux PP . Activation and function of the MAPKs and their substrates, the MAPK-activated protein kinases. Microbiol Mol Biol Rev. (2011) 75:50–83. doi: 10.1128/MMBR.00031-10 21372320 PMC3063353

[52] Hu X Li J Fu M Zhao X Wang W . The JAK/STAT signaling pathway: from bench to clinic. Signal Transduct Target Ther. (2021) 6:402. doi: 10.1038/s41392-021-00791-1 34824210 PMC8617206

[53] Takatori H Kawashima H Suzuki K Nakajima H . Role of p53 in systemic autoimmune diseases. Crit Rev Immunol. (2014) 34:509–16. doi: 10.1615/critrevimmunol.2014012193 25597313

[54] Hartsock A Nelson WJ . Adherens and tight junctions: structure, function and connections to the actin cytoskeleton. Biochim Biophys Acta. (2008) 1778:660–9. doi: 10.1016/j.bbamem.2007.07.012 17854762 PMC2682436

[55] Ramasamy A Mohan C . Molecular and cellular mediators of renal fibrosis in lupus nephritis. Int J Mol Sci. (2025) 26:2621. doi: 10.3390/ijms26062621 40141260 PMC11942537

[56] Zhang F Wu L Qian J Qu B Xia S La T . Identification of the long noncoding RNA NEAT1 as a novel inflammatory regulator acting through MAPK pathway in human lupus. J Autoimmun. (2016) 75:96–104. doi: 10.1016/j.jaut.2016.07.012 27481557

[57] Cai Z Zhang S Wu P Ren Q Wei P Hong M . A novel potential target of IL-35-regulated JAK/STAT signaling pathway in lupus nephritis. Clin Transl Med. (2021) 11:e309. doi: 10.1002/ctm2.309 33634995 PMC7851357

[58] Dong J Wang L Zhao L Pan L Zhang Y . Effect of plasma exosomes on endothelial cell tight junction proteins in SLE patients with immune thrombocytopenia. Clin Rheumatol. (2021) 40:3273–8. doi: 10.1007/s10067-021-05651-5 33599860

