# Supplementary material for: Circulating long non-coding RNAs as diagnostic markers of lupus nephritis and 5-year follow-up disease flares
Source: Front Immunol. 2026 Aug 4;17:1716331. doi: 10.3389/fimmu.2026.1716331 (PMC13481647; doi:10.3389/fimmu.2026.1716331)
Supplement: Supplementary file 1 [file DataSheet1.docx]

Supplementary Material

Circulating long non coding RNAs as diagnostic markers of lupus nephritis and 5-year follow-up disease flares

Authors: Ana Flores-Chova^1,†^, Olga Martinez-Arroyo^1,†^, Marta Mendez-Debaets^1^, Lesley Escriva^1^, Ana Ortega^1,2,^*, Maria J Fornera^1,3,4,‡^, Raquel Cortes^1,‡,*^

Affilliations:

^1^Cardiometabolic and Renal Risk Research Group, INCLIVA Biomedical Research Institute, Valencia, Spain

**^2^CIBERCV (CIBER of Cardiovascular diseases), Madrid, Spain**

^3^ Medicine Unit, Hospital Clinico Universitario de Valencia, Valencia, Spain

^4^Department of Medicine, University of Valencia, Valencia, Spain

^†^These authors have contributed equally to this work and share first authorship

^‡^These authors have contributed equally to this work and share last authorship

^*^Corresponding Authors: Ana Ortega (aortega@incliva.es) and Raquel Cortes (raquel.cortes@uv.es)

# 1 Supplementary Tables

# Table S1. Primers used in the RT-qPCR for mRNA analysis

| **lncRNA** | **Forward 5' → 3'** | **Reverse 3' → 5'** |
| --- | --- | --- |
| AL139317.5-L | TCCCAGGAGACATTTGGCAA | ACCGATCAGAAAATAGCCTGTG |
| AC019080.1-L | GTTTCACCATGTTGCCCAGG | GGCACCTTCTGTAATCCTAGCA |
| LINC00708-L | CTTGGTGCTGAGGACATTGT | GGTTTGCAATACTTTTCCACGT |
| AC087392.4-L | TGAGGCAATGAGGAAACCAGA | ATGAGGATGACGGGAGGAGT |
| AC239868.1-R | TTTTAACCGCTACGCCACAG | TTCCCTTAGCCTCAGTGCAA |
| LATS2-AS1-L | TAATTCGCACTCTTGCTGGGA | ACTAAATTGGCCTCCCAAAGC |
| LINC02185_R | TGTGAAGGCAAAAGATGAACCA | GAACAGTCTGCTTTTCTCTCCG |
| ACTB | TGGAGAAAATCTGGCACCAC | CATGATCTGGGTCATCTTCTCG |

# Table S2. Correlations between lncRNA levels with disease related features.

| lncRNA |  | dsDNA | C3 | C4 | SLEDAI index | UAE/u-creatinine |
| --- | --- | --- | --- | --- | --- | --- |
| AL139317.5 | Spearman coefficient | 0.077 | 0-.090 | -0.037 | 0.113 | **0.464** |
|  | p value | 0.474 | 0.401 | 0.730 | 0.305 | **0.001** |
| LINC02185 | Spearman coefficient | 0.051 | **-0.302** | -0.152 | **0.308** | **0.453** |
|  | p value | 0.632 | **0.004** | 0.154 | **0.004** | **0.002** |
| AC019080.1 | Spearman coefficient | -0.026 | -0.103 | -0.140 | 0.175 | **0.260** |
|  | p value | 0.809 | 0.336 | 0.188 | 0.109 | **0.035** |
| LINC00708 | Spearman coefficient | 0.201 | -0.017 | -0.049 | **0.236** | **0.293** |
|  | p value | 0.059 | 0.875 | 0.644 | **0.030** | **0.017** |
| AC239868.1 | Spearman coefficient | 0.095 | -0.133 | -0.115 | **0.238** | **0.366** |
|  | p value | 0.376 | 0.211 | 0.281 | **0.028** | **0.005** |
| AC087392.4 | Spearman coefficient | -0.045 | 0.006 | -0.031 | -0.023 | **0.275** |
|  | p value | 0.673 | 0.955 | 0.769 | 0.833 | **0.019** |
| LATS2_AS1 | Spearman coefficient | **0.236** | -0.024 | -0.140 | 0.120 | **0.291** |
|  | p value | **0.026** | 0.825 | 0.187 | 0.274 | **0.018** |

C3: complement C3; C4: complement C4; dsDNA: double strand DNA; Text in bold shows the significant correlations.

# Table S3. Receiver-operating characteristic curve of circulating lncRNAs for detecting systemic lupus erythematosus.

| **lncRNA** | **AUC** | **95% CI** | **P value** | **SS** | **SP** | **PPV** | **NPV** |
| --- | --- | --- | --- | --- | --- | --- | --- |
| AC019080.1 | 0.797 | 0.716-0.877 | 0.0001 | 57 | 88 | 95 | 35 |
| AL139317.5 | 0.877 | 0.817-0.937 | 0.0001 | 80 | 88 | 94 | 35 |
| LINC02185 | 0.862 | 0.791-0.933 | 0.0001 | 59 | 92 | 97 | 37 |
| LINC00708 | 0.775 | 0.677-0.873 | 0.0001 | 46 | 88 | 94 | 30 |
| AC087392.4 | 0.773 | 0.684-0.863 | 0.0001 | 55 | 84 | 93 | 30 |
| LATS2.AS1 | 0.701 | 0.605-0.797 | 0.001 | 42 | 92 | 95 | 29 |
| AC239868.1 | 0.855 | 0.790-0.920 | 0.0001 | 77 | 84 | 95 | 49 |
| 7-Signature | 0.978 | 0.954-1.000 | 0.0001 | 91 | 92 | 97 | 72 |

# Sensitivities, specificities, and predictive values (%) for the diagnostic of lupus nephritis (cut-off point fold change ≥ 2.0 for each lncRNA). AUC, area under the curve; CI, confidence interval; NPV, negative predictive value; PPV, predictive positive value; SP, specificity; SS, sensitivity.

# Table S4. Receiver-operating characteristic curve of circulating lncRNAs for detecting lupus nephritis.

| **lncRNA** | **AUC** | **95% CI** | **P value** | **SS** | **SP** | **PPV** | **NPV** |
| --- | --- | --- | --- | --- | --- | --- | --- |
| AC019080.1 | 0.911 | 0.821-1.000 | 0.0001 | 70 | 96 | 94 | 75 |
| AL139317.5 | 0.913 | 0.826-1.000 | 0.0001 | 78 | 92 | 95 | 83 |
| LINC02185 | 0.877 | 0.770-0.983 | 0.0001 | 61 | 96 | 93 | 73 |
| LINC00708 | 0.821 | 0.698-0.944 | 0.0001 | 52 | 96 | 93 | 71 |
| AC087392.4 | 0.821 | 0.701-0.941 | 0.0001 | 61 | 84 | 78 | 70 |
| LATS2.AS1 | 0.748 | 0.602-0.894 | 0.003 | 52 | 92 | 86 | 68 |
| AC239868.1 | 0.858 | 0.742-0.974 | 0.0001 | 74 | 92 | 89 | 79 |
| 7-Signature | 1.000 | 1.000-1.000 | 0.0001 | 100 | 100 | 100 | 100 |

# Sensitivities, specificities, and predictive values (%) for the diagnostic of lupus nephritis (cut-off point fold change ≥ 2.0 for each lncRNA). AUC, area under the curve; CI, confidence interval; NPV, negative predictive value; PPV, predictive positive value; SP, specificity; SS, sensitivity.

**Table S5**. Statistical comparison of AUC of the combined signatures versus each individual lncRNA for SLE and LN.

|  | **SLE** | **LN** |
| --- | --- | --- |
|  | **P-value** | **P-value** |
| 7-signature vs AL139317.5 | ***0.002378641*** | ***0.048010811*** |
| 7-signature vs AC019080.1 | ***2.26638E-05*** | ***0.043101273*** |
| 7-signature vs LINC02185 | ***0.002236624*** | ***0.022739821*** |
| 7-signature vs LINC00708 | ***7.88422E-05*** | ***0.004493427*** |
| 7-signature vs AC239868.1 | ***0.00046028*** | ***0.016093877*** |
| 7-signature vs AC087392.4 | ***1.61633E-05*** | ***0.00334165*** |
| 7-signature vs LATS2_AS1 | ***4.0011E-08*** | ***0.000779425*** |

LN, lupus nephritis; SLE, Systemic lupus erythematosus.

**Table S6**. Statistical comparison of AUC of the combined lncRNA signatures for SLE and LN discrimination.

|  | **SLE** | | | | | **LN** | | | | |
| --- | --- | --- | --- | --- | --- | --- | --- | --- | --- | --- |
|  | **AUC** | **p-value** | **95% CI** | | **p-value** | **AUC** | **p-value** | **95% CI** | | **p-value** |
| **SLE** |  |  | **Lower limit** | **Upper limit** |  |  |  | **Lower limit** | **Upper limit** |  |
| 7-signature | 0.978 | 0.000 | 0.954 | 1 |  | 1.00 | 0.000 | 1.000 | 1.000 |  |
| 6-signature | 0.950 | 0.000 | 0.917 | 0.989 | *0.195* | 1.00 | 0.000 | 1.000 | 1.000 | 1.000 |
| 5-signature | 0.948 | 0.000 | 0.913 | 0.987 | *0.181* | 1.00 | 0.000 | 1.000 | 1.000 | 1.000 |
| 4-signature | 0.947 | 0.000 | 0.907 | 0.986 | *0.183* | 0.991 | 0.000 | 0.972 | 1.000 | 0.368 |
| 3-signature | 0.931 | 0.000 | 0.905 | 0.985 | ***0.047*** | 0.944 | 0.000 | 0.862 | 1.000 | ***0.045*** |
| 2-signature | 0.898 | 0.000 | 0.853 | 0.963 | ***0.008*** | 0.943 | 0.000 | 0.860 | 1.000 | ***0.036*** |

signature-7: AL139317.5, AC019080.1, LINC02185, LINC00708, AC239868.1, AC087392.4, and LATS2-AS1; signature-6: AL139317.5, AC019080.1, LINC02185, LINC00708, AC239868.1, AC087392.4; signature-5: AL139317.5, AC019080.1, LINC02185, LINC00708, AC239868.1, signature-4: AL139317.5, AC019080.1, LINC02185, AC239868.1, signature-3: AL139317.5, LINC02185, AC239868.1, signature-2: AL139317.5, AC239868.1. AUC, area under the curve; CI, confidence interval; LN, lupus nephritis; SLE, systemic lupus erythematosus.

**Table S7.** Baseline treatment of patients according to lupus flare status at 5-year follow-up.

|  | **No flare**  **(n=62)** | **Flare**  **(n=34)** | **p-value** |
| --- | --- | --- | --- |
| Baseline treatment (%) |  |  |  |
| Hydroxychloroquine | 64.5 | 65.6 | n.s |
| Azathioprine | 17.7 | 21.2 | n.s |
| Prednisone | 40.3 | 56.3 | n.s |
| Deflazacort | 11.3 | 9.4 | n.s |
| Belimumab or Rituximab | 3.2 | 12.5 | n.s |
| Metotrexate | 4.8 | 9.1 | n.s |
| Mycophenolate Mofetil | 3.2 | 6.1 | n.s |

**Table S8**. Statistical comparison of AUC of the combined lncRNA signatures for lupus flares prediction.

|  | **AUC** | **p-value** | **95% CI** | | **p-value** |
| --- | --- | --- | --- | --- | --- |
| **Flares** |  |  | **Lower limit** | **Upper limit** |  |
| 6-signature | 0.853 | 0.000 | 0.775 | 0.930 |  |
| 5-signature | 0.851 | 0.000 | 0.771 | 0.932 | *0.978987604* |
| 4-signature | 0.815 | 0.000 | 0.731 | 0.899 | 0.51550179 |
| 3-signature | 0.733 | 0.000 | 0.643 | 0.823 | ***0.048177269*** |
| 2-signature | 0.726 | 0.000 | 0.632 | 0.820 | ***0.041376703*** |

signature-6: AL139317.5, AC019080.1, LINC02185, LINC00708, AC239868.1, LATS2-AS1; signature-5: AL139317.5, LINC02185, LINC00708, AC239868.1, LATS2-AS1; signature-4: AL139317.5, LINC02185, LINC00708, AC239868.1; signature-3: AL139317.5, LINC02185, AC239868.1; signature-2: AL139317.5, AC239868.1. AUC, area under the curve; CI, confidence interval.

**Table S9. Multivariate Cox regression analysis for predicting 5-year follow-up flares.**

| **Variable** | **p value** | **HR** | **95.0% CI HR** | |
| --- | --- | --- | --- | --- |
|  |  |  | Lower limit | Upper limit |
| 4-lncRNA signature | **0.005** | **4.748** | 1.600 | 14.088 |
| Gender (Female) | 0.563 | 0.610 | 0.114 | 3.251 |
| Age | 0.611 | 0.989 | 0.947 | 1.033 |
| SLEDAI index | 0.358 | 1.048 | 0.948 | 1.158 |
| Azathioprine | 0.474 | 0.624 | 0.172 | 2.268 |
| Hidroxychloroquine | 0.540 | 0.696 | 0.218 | 2.218 |
| Prednisone | 0.871 | 0.908 | 0.284 | 2.903 |
| Deflazacort | 0.924 | 0.919 | 0.164 | 5.160 |
| Belimumab | 0.006 | 0.105 | 0.021 | 0.529 |
| Metotrexate | 0.431 | 0.538 | 0.115 | 2.518 |
| Mycophenolate mofetil | 0.738 | 1.504 | 0.137 | 16.458 |
| Ciclophosphamine | 0.290 | 0.532 | 0.166 | 1.712 |
| C3 (<90 mg/dL) | 0.290 | 0.532 | 0.166 | 1.712 |
| C4 (<10 mg/dL) | 0.944 | 0.955 | 0.261 | 3.490 |
| dsDNA (>35 UI/mL) | 0.540 | 0.696 | 0.218 | 2.218 |
| LN diagnostic | 0.631 | 1.377 | 0.374 | 5.073 |
| Duration disease (years) | 0.700 | 0.987 | 0.926 | 1.053 |

C3: complement C3; C4: complement C4; dsDNA: double strand DNA; LN: lupus nephritis; Systemic Lupus Erythematosus Disease Activity Index. Classification of categorical variables in the multivariate Cox regression analysis: Categorical dsDNA: cut-off point 35 UI/mL; Categorical C3, cut-off point 90 mg/dL; Categorical C4, cut-off point 10 mg/dL; Categorical dsDNA, cut-off point 35; 4-lncRNA signature (optimal cut-off of ROC curve)

# Table S10. LncRNA-miRNA interactions identified by LncBase Diana Tools.

| **lncRNA** | **miRNA targets** |
| --- | --- |
| AC019080.1 | hsa-miR-103a-3p |
|  | hsa-miR-107 |
|  | hsa-miR-15a-5p |
|  | hsa-miR-15b-5p |
|  | hsa-miR-16-5p |
|  | hsa-miR-195-5p |
|  | hsa-miR-200c-3p |
|  | hsa-miR-210-3p |
|  | hsa-miR-218-5p |
|  | hsa-miR-27a-3p |
|  | hsa-miR-93-5p |
| AC239868.1 | hsa-let-7a-5p |
|  | hsa-let-7e-5p |
|  | hsa-let-7f-5p |
|  | hsa-let-7g-5p |
|  | hsa-let-7i-5p |
|  | hsa-miR-106a-5p |
|  | hsa-miR-106b-5p |
|  | hsa-miR-15a-5p |
|  | hsa-miR-15b-5p |
|  | hsa-miR-17-5p |
|  | hsa-miR-18a-5p |
|  | hsa-miR-20a-5p |
|  | hsa-miR-28-5p |
|  | hsa-miR-34a-5p |
|  | hsa-miR-423-5p |
|  | hsa-miR-424-5p |
|  | hsa-miR-7-5p |
|  | hsa-miR-708-5p |
|  | hsa-miR-9-5p |
|  | hsa-miR-93-5p |
|  | hsa-miR-98-5p |
|  | kshv-miR-K12-3-3p |
|  | kshv-miR-K12-5-3p |
| AL139317.5 | hsa-let-7e-5p |
|  | hsa-let-7i-5p |
|  | hsa-miR-17-5p |
|  | hsa-miR-302a-3p |
|  | hsa-miR-302b-3p |
|  | hsa-miR-302c-3p |
|  | hsa-miR-302d-3p |
|  | hsa-miR-302e |
|  | hsa-miR-34a-5p |
|  | hsa-miR-98-5p |

**2. Supplementary Figures**

**Figure S1.** Validated lncRNA profile in SLE and LN patients by qRT-PCR. Violin plot graph of circulating expression levels of lncRNAs selected as potential biomarkers for the detection of SLE and LN. Comparison between patients with (LN, n=23) or without lupus nephritis (nLN, n=73) and healthy controls (CNT, n=25). Data were represented as the median (line), quartiles (dotted lines), maximum and minimum and the probability density of the data at different values. Data was compared using Mann-Whitney U test. *p < 0.05, **p < 0.001, ***p<0.0001,

**Figure S2.** Diagnostic value of lncRNA profile and UAE in LN patients. A. Receiver operating characteristic (ROC) curves of the combined 7-lncRNA panel and UAE/urinary creatinine for the detection of LN. AUC: area under the curve. LN: lupus nephritis.
